# Dosimetric verification of IMPT using a commercial heterogeneous phantom

**DOI:** 10.1002/acm2.12535

**Published:** 2019-01-23

**Authors:** Keisuke Yasui, Toshiyuki Toshito, Chihiro Omachi, Kensuke Hayashi, Hideto Kinou, Masaki Katsurada, Naoki Hayashi, Hiroyuki Ogino

**Affiliations:** ^1^ Faculty of Radiological Technology School of Health Sciences Fujita Health University 1‐98 Dengakugakubo, Kutsukake‐cho Toyoake Aichi 470‐1192 Japan; ^2^ Nagoya Proton Therapy Center Nagoya City West Medical Center 1‐1‐1, Hirate‐cho, Kita‐ku Nagoya Aichi 462‐8508 Japan

**Keywords:** chamber measurement, dosimetric verification, IMPT, spot scanning

## Abstract

The purpose of this study was to propose a verification method and results of intensity‐modulated proton therapy (IMPT), using a commercially available heterogeneous phantom. We used a simple simulated head and neck and prostate phantom. An ionization chamber and radiochromic film were used for measurements of absolute dose and relative dose distribution. The measured doses were compared with calculated doses using a treatment planning system. We defined the uncertainty of the measurement point of the ionization chamber due to the effective point of the chamber and mechanical setup error as 2 mm and estimated the dose variation base on a 2 mm error. We prepared a HU‐relative stopping power conversion table and fluence correction factor that were specific to the heterogeneous phantom. The fluence correction factor was determined as a function of depth and was obtained from the ratio of the doses in water and in the phantom at the same effective depths. In the simulated prostate plan, composite doses of measurements and calculations agreed within ±1.3% and the maximum local dose differences of each field were 10.0%. Composite doses in the simulated head and neck plan agreed within 4.0% and the maximum local dose difference for each field was 12.0%. The dose difference for each field came within 2% when taking the measurement uncertainty into consideration. In the composite plan, the maximum dose uncertainty was estimated as 4.0% in the simulated prostate plan and 5.8% in the simulated head and neck plan. Film measurements showed good agreement, with more than 92.5% of points passing a gamma value (3%/3 mm). From these results, the heterogeneous phantom should be useful for verification of IMPT by using a phantom‐specific HU‐relative stopping power conversion, fluence correction factor, and dose error estimation due to the effective point of the chamber.

## 1 INTRODUCTION

In recent years, proton beams have become widely used for the treatment of various types of cancer. Active scanning is one of the delivery techniques for proton therapy.^1^ The active scanning method moves a spot along the longitudinal and horizontal axis and uses different energy beams to create three‐dimensional dose distributions. There are two different delivery methods for scanning proton therapy; the first is single field uniform dose (SFUD)^2^ and the second is intensity‐modulated proton therapy (IMPT).^3^, ^4^ IMPT is more flexible than SFUD and, in general, can deliver a more conformal prescribed dose to the target with lower dose to organs at risks (OARs). IMPT is more sensitive to the uncertainty due to the proton range and mechanical errors than SFUD because IMPT delivers a nonuniform dose distribution to each field. Since the total dose distributions of IMPT are created by summation of some nonuniform dose distributions, it can potentially cause unexpected dramatically hot or cold spots by the combination of inhomogeneity of the human body, machine variations, range uncertainty, and accuracy of the beam modeling or the dose calculation algorithm.^5^, ^6^ From this perspective, accurate commissioning, quality assurance (QA) including machine validations and verification of the treatment planning system are fundamental to implementing high‐quality IMPT. Heterogeneous phantoms have been effectively used to evaluate the total dose including accuracy of dose calculation and machine variations, and to identify problems that are not revealed by the homogeneous phantom measurements. For this reason, anthropomorphic heterogeneous phantoms are used for the verification of intensity‐modulated radiotherapy^7^, ^8^ and also used with a thermoluminescent dosimeter (TLD) dosimeter and radiochromic film to verify scattering, pencil beam and uniform scanning plans.^9^, ^10^ Furthermore, the results of patient‐specific QA of IMPT in some sites using various techniques were excellent.^11^, ^12^, ^13^, ^14^, ^15^ However, few studies have focused on the verification for IMPT using commercially available system, especially absolute dose measurement using heterogeneous phantom and ionization chamber. The paper by Taylor et al.^10^ reported the summarization for clinical trial credentialing of scattered and scanning proton beam using TLD dosimeter, radiochromic film, several types of IROC's anthropomorphic proton phantoms, and commercial or in‐house treatment planning systems (TPSs) that use pencil beam or Monte Carlo‐based algorithms. In this study, we report methods and results of verification for the IMPT using a commercially available simple heterogeneous phantom, new TPS, an ionization chamber, and radiochromic film. Our new TPS is the first system that uses the triple Gaussian (TG) model‐based pencil beam algorithm. To the best of our knowledge, this is the first report of dosimetric verification of the new TG model‐based TPS using commercial heterogeneous phantom and to analyze uncertainties of effective point for the absolute dose measurement using ionization chamber for IMPT.

## 2 MATERIALS AND METHODS

### 2.A Depth and fluence correction for heterogeneous phantom

**Figure 1:**
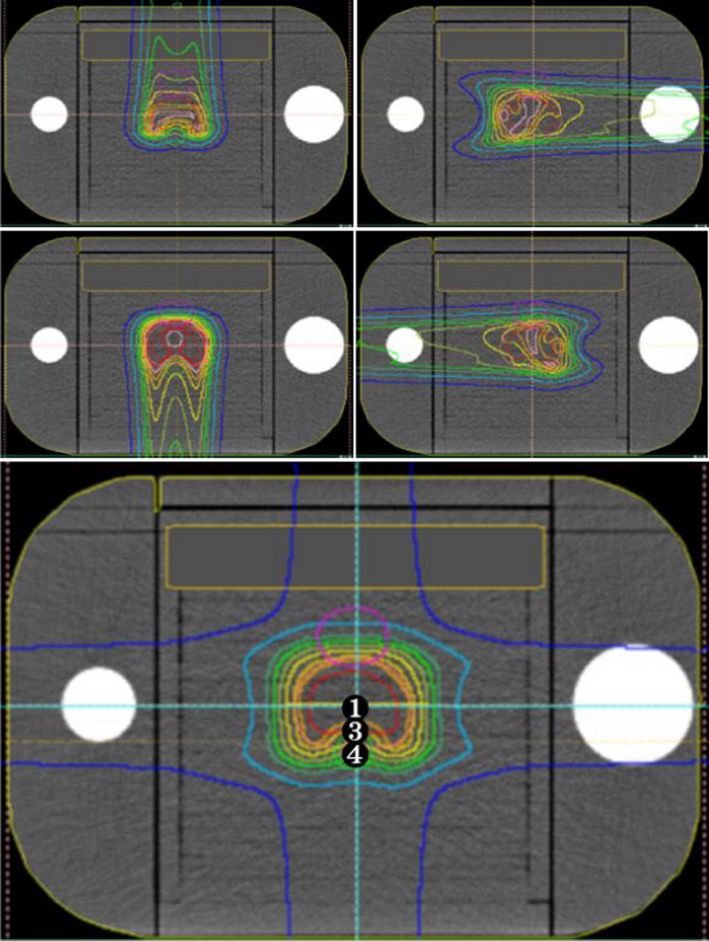
Measurement points and dose distributions of each field and accumulated dose of simulated prostate plan. Circled numbers shown in the accumulated image are measurement dose points. Measurement point 2 was the maximum dose point and overlapping in number 1.

In this study, we used the RT‐3000‐New phantom (RT‐3000, R‐Tech.inc, Tokyo, Japan) that simulates the simple head and neck (HN) and prostate using simulated‐bone material. RT‐3000 is made from acrylonitrile‐butadiene‐styrene (ABS) and artificial bone material. Diameters of the simulated‐bone material for prostate are 2.5 cm (small) and 4.2 cm (large) and for HN is 1.0 cm, as shown in Fig. 1, 2. In our clinical practice, relative stopping powers (RSPs) were calculated with human tissues as described in ICRU reports 44^16^ and 46^17^ using the stoichiometric calibration proposed by Schneider et al.^18^ However, an error of several percent occurs when clinical computed tomography (CT) values (Hounsfield Units: HU) — RSPs conversion table is applied to the material of RT‐3000 phantom. So to use RT‐3000 phantom for the verification of IMPT, we created a phantom‐specific HU‐RSP conversion table using measured RSPs and CT values following the method proposed by Grant et al.^19^ We measured RSP of water equivalent materials (soft‐tissue phantom) and bone materials (bone phantom) of various thicknesses. A 221.4 MeV proton beam, having a penetration depth of 30.6 cm, was used to measure the change in depth of the distal 90% dose. To create the phantom‐specific HU‐RSP conversion table, we used the average rate of changes in depth and thicknesses of each material. The fluence correction is another important factor for absolute dose measurement using a solid phantom.^20^ In this study, we obtained the fluence correction factor as the ratio of measured dose in the water and that in the soft‐tissue phantom material at the same effective depths. Subsequently, an approximate linear formula was derived as the fluence correction factor. The fluence correction for bone phantom is not considered in this paper. The scanned field size was 10 × 10 cm^2^, and the energies and SOBPs used were adapted to each measurement depth. The scanning patterns and measurement depths are summarized in Table 1. To measure at 3 cm depth, we used an energy absorber that had 4 cm water equivalent thickness because the minimum penetration depth of our system was 4 cm. The details on energy absorber will be mentioned in the next section.

**Figure 2:**
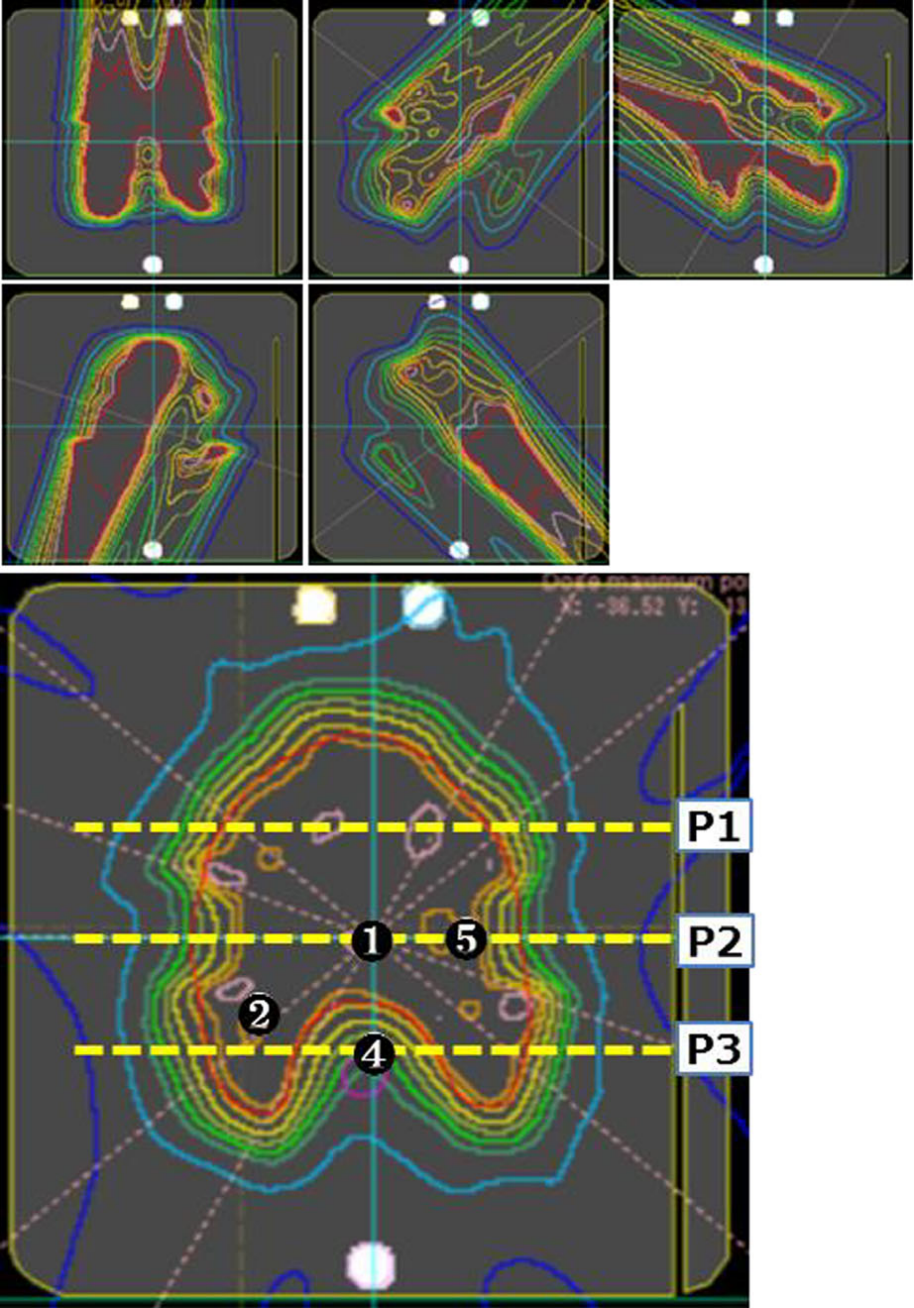
Measurement points and dose distributions of each field and accumulated dose of simulated HN plan. Circled numbers shown in the accumulated image are measurement dose points and dashed lines (P1–P3) indicate the planes of film measurements. Measurement point number 3 was overlapping in number 1.

**Table 1:** Scanning patterns and measurement depths to measure fluence correction factor

Phantom thickness (cm)	Water depth (cm)	Energy (MeV)	Range (cm)	SOBP width (cm)	MU
3	3.05	111.4–82.2	4.9	4	207.40
5	5.08	103.1–71.6	7.9	4	195.93
10	10.15	130.5–104.5	12.1	4	170.89
15	15.23	175.7–132.3	20.5	10	191.48
20	20.30	200.5–152.6	25.8	10	194.13
25	25.38	221.4–178.2	30.6	10	187.07

### 2.B Treatment planning and proton delivery system

The TPS used in this study was VQA (ver 3.0.1 Hitachi Ltd., Tokyo, Japan), a commercially available TPS in Japan. This is the first system that uses the TG model for the dose calculation. The TG model shows distinct difference with other low‐dose kernel models in the high energy region where the influence of the secondary particles produced by nuclear interactions in water is increased. In the typical volumetric irradiation, the TG model reported better agreement to the measurement value especially small or large fields and narrow SOBP width condition.^21^ The structures, mock clinical target volume (CTV) and OARs of prostate and HN, and dose constraints were adopted from AAPM Task Group‐119^22^ that has produced quantitative confidence limits as baseline expectation values for IMRT commissioning. We used the worst case optimization,^23^ the parameters of which were 3 mm for each direction and 3.5% for range uncertainty. The scanning delivery system used in this study was installed in Nagoya Proton Therapy Center. The synchrotron can produce 95 proton beams energies having water penetration distances from 40 to 306 mm. The maximum spot size (1*σ*) in air at the isocenter plane is 13.8 mm, and the minimum is 4.7 mm.^24^, ^25^ To irradiate shallow regions less than 40 mm, we used the energy absorber that had 40 mm water equivalent thickness. The energy absorber can be attached to the beam nozzle and can be used with patient‐specific aperture.^26^ The spot size of minimum range with energy absorber was 26.7 mm. In this study, we use energy absorber to measure fluence correction factor at 3 cm depth. The energies used in verification plan were from 84.7 to 187.7 MeV for HN planning and from 100.2 MeV to 205.9 MeV for prostate planning. In a clinical setting, we use two parallel‐opposed SFUD fields for prostate treatment; however, the simulated prostate plan was created with a 4‐fields IMPT plan in this study. The HN plan was created with five fields.

### 2.C Experimental measurements

An ionization chamber (3D‐PinPopint chamber, PTW30016) and a radiochromic film (Gafchromic EBT3 film, ISP) were used for absolute and dose distribution measurement. The absolute dose measurement involves uncertainties such as effective points of the ionization chamber, setup error, and variations of the mechanical isocenter of a gantry rotation. In the measurement of absolute dose for proton beam, the uncertainty of the effective point is particularly an important issue because proton dose distributions have a gradient to the depth direction unlike x‐ray dose distributions. Hence, the effective measurement point of the cylindrical ionization chamber should be considered to each gantry angle and the dose uncertainty due to the effective point is not able to estimate by the total dose distribution. The effective measurement point of the cylindrical ionization chamber in proton therapy has been investigated in several studies, and was roughly 1 mm for the PTW 31016 chamber.^26^, ^27^, ^28^ The uncertainty of the mechanical isocenter of gantry rotation of the our system has less than 1 mm accuracy.^29^ In this report, we have dealt with the error of the measurement point up to 2 mm to the source direction and 1 mm to other directions for each gantry angle, and dose uncertainties were presumed from calculation results of each field dose of TPS. In the simulated prostate plan, the ionization chamber was located at the isocenter, near the rectum region and maximum dose point. In the simulated HN plan, the ionization chamber was located at the isocenter, near to OARs and hot spots. EBT3 films were cut to fit the RT‐3000 phantom and inserted into the phantom on three planes. The dose calibration curve creation and film scanning were performed as reported by Zhao and Das^30^ and we obtained a calibration curve at the SOBP center using proton beams from 221.4 MeV to 178.2 MeV that was SOBP 10 cm. Each film was scanned using commercial flatbed scanner (ES‐10000G, EPSON) and analyzed using dosimetry evaluation software (Film Analysis and VeriSoft, PTW). We analyzed the film dose by two‐dimensional (2D)‐to‐2D Gamma index calculation.^32^ The measurement points, measurement planes, and dose distributions are shown in Figs. 1 and 2.

## 3 RESULTS AND DISCUSSION

### 3.A Depth and fluence correction for heterogeneous phantom

**Figure 3:**
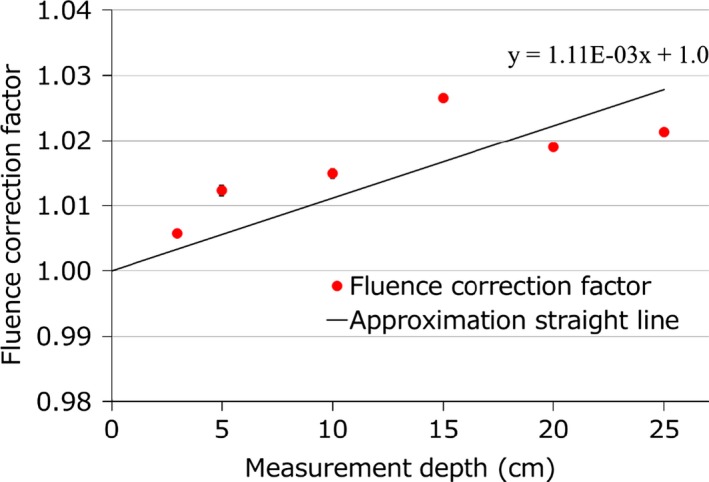
Experimental fluence correction factor vs measurement depth. Line shows the linear approximation curve of the fluence correction factor passing through 1.0 at 0 cm depth. The formula shown in the figure is the linear approximation formula of the fluence correction factor.

Table 2 shows the results of the RSP measurements. HU values showed some dispersion of about 40 HU. In this study, we used RSP values that correspond to each material. The RSPs of soft‐tissue phantom were different by less than 2% from the clinical RSP, although the bone phantom that simulated bone was different by more than 30%. Consequently, the RT‐3000 phantom required a phantom‐specific HU‐RSP conversion table especially for the bone phantom because the phantom was made from artificial materials. The uncertainty of the RSP for the RT‐3000 phantom was estimated as 0.45% from the results of multiple measurements of the materials and this uncertainty was sufficiently small. Figure 3 shows the measured results for the fluence correction factor. We adopted a linear approximation formula method to correct measurement values by depth. These results closely resemble those of a previous study^20^ that were obtained with a nonmodulated 191 MeV beam, a PMMA, and a Markus‐type plane‐parallel ionization chamber. We assume that the dose distributions were formed by the convolution of nonmodulated proton beams, so the fluence scaling factor of our scanning method was closer to the nonmodulated result of the previous study. However, the fluence correction needs further investigation because it is affected by various factors such as beam energy, measurement point in the irradiated volume, field size, and scanning pattern. In this study, the maximum difference of the fluence correction factor between experimental measurements and the linear approximation formula was 1.0% at 15 cm depth.

**Table 2:** Variation of HU, average thickness (tm), range variation, calculate and measurement RSP of each simulated material used in the heterogeneous phantom. The calculate RSP was determined by the HU‐RSP conversion table used in clinical

Simulated materials	HU	Average tm (mm)	Average range variation (mm)	Measurement RSP	Calculate RSP
Soft‐tissue	−20 to −60	12.93	13.16	1.015	0.980
Bone	920 to 970	19.96	35.73	1.789	1.492

### 3.B Dosimetric verification for IMPT

**Figure 4:**
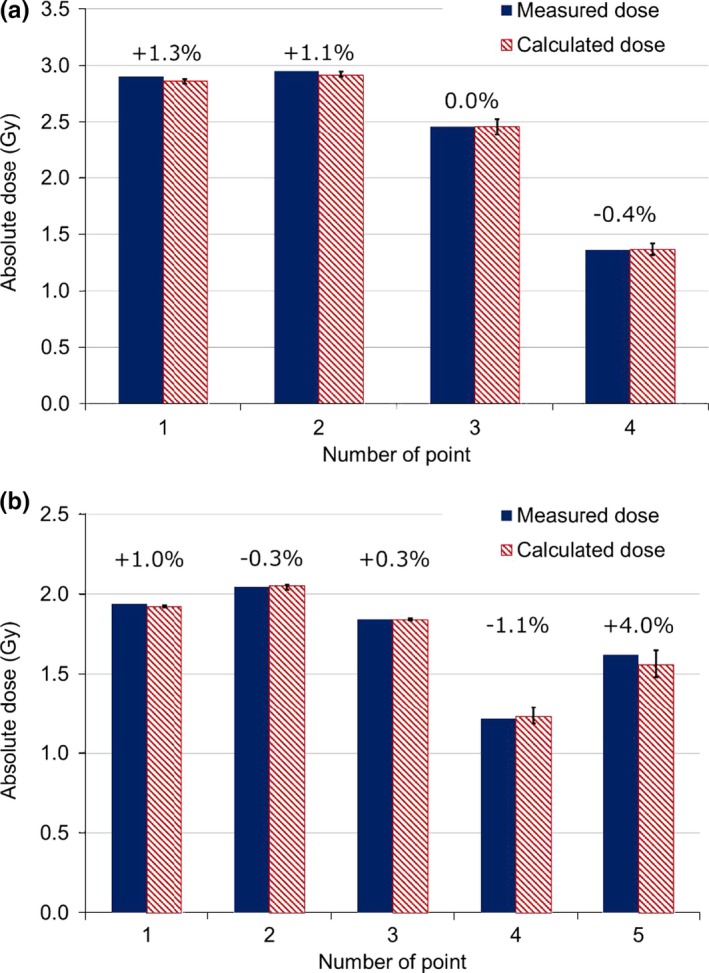
Comparison between measured and calculated doses for the simulated plan: [(measured dose) − (plan dose)]/plan dose × 100 (%). Upper figure showed prostate plan (a), Lower figure showed HN plan (b). Local dose differences are shown above each bar. Error bars indicate dose uncertainties due to estimated uncertainty.

Figure 4 shows local dose differences between chamber measurements and calculations of each point of composite dose in the prostate and the HN plans. In the simulated prostate plan, composite doses of measurements and calculations agreed within ±1.3% at all measurement points. The maximum local dose difference of each field dose was +10.0% at the steepest dose gradient and low dose point. Composite doses in the simulated HN plan agreed within ±4.0% and the maximum local difference of each field dose was +12.0%. The error bars in Fig. 4 represent total dose uncertainties due to the 2 mm measurement point error to the source direction and the 1 mm error to other directions for each gantry angle as described in Materials and Methods. Given this uncertainty of the measurement point, the maximum dose uncertainty was estimated as 3.9% for point 3 of the simulated prostate plan. The maximum dose uncertainty of the simulated HN plan was estimated as 5.8% at point 5. The point 5 was set near the simulated parotid, therefore, that was in the steep dose gradient region and have large dose uncertainty as shown in Figs. 2 and 4. The dose difference of each field dose was within 2% at all measurement points taking the measurement uncertainties into consideration. Dose differences of the composite dose agreed within 0.7% considering the measurement uncertainties shown in Fig. 4. From these results, verification using the simple heterogeneous phantom was useful; however, it remains necessary to consider measurement point uncertainties because some points of IMPT fields may have steep gradients in various directions.

**Figure 5:**
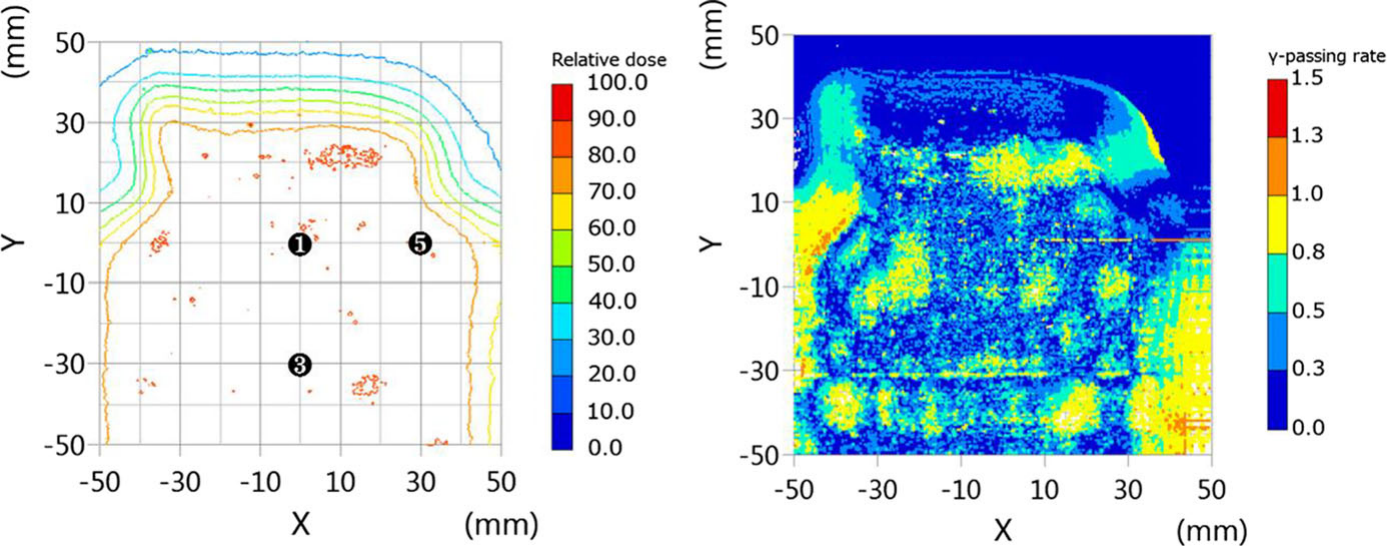
Examples of dose distributions (left) and results of gamma analysis (right) of P2 plane. Numbers in figure showed absolute dose measurement points indicated in Fig. 2.

The results of gamma analysis for film measurements showed good agreement at 95.7% (isocenter plane: P1), 96.0% (40 mm anterior from isocenter plane: P2) and 92.5%, and (30 mm posterior from isocenter plane: P3) of measurement points passed 3%/3 mm criteria (threshold dose level = 30%). Figure 5 shows the dose distributions and results of gamma analysis of P2. The number of points with gamma values greater than 1.0 was small. These errors were due to uncertainties of the film measurement and calculation accuracy of TPS. The film measurement of proton therapy has uncertainties such as Linear Energy Transfer (LET) dependence and noise. Furthermore, when the using uniform phantom, the largest dose difference in the typical volumetric irradiation of our TPS is 1.3% at the shallow region of the small field.^21^ In this study, the calculation dose error was assumed to increase because we use IMPT field and heterogeneous phantom. However, the dose differences of these error points were within 5%. Previous studies have reported that radiochromic film shows under‐response of about 10% at the Bragg peak region and a variation in film sensitivity due to LET of 5%^30^, ^31^, ^32^, ^33^, ^34^; therefore, results of this study showed sufficiently good. The reason for the good gamma pass rate and dose agreement within 5% were presumed to be that the average LET of IMPT HN plan was almost the same as the LET of the calibration depth.

## 4 CONCLUSION

Herein, we reported dosimetric verification methods and results for the IMPT with new TG model‐based TPS using a commercially available simple heterogeneous phantom, an ionization chamber, and radiochromic film. Considering various uncertainties, the measured and calculated doses showed good agreement. Our results demonstrated that heterogeneous phantoms are useful for verification of IMPT by using phantom‐specific HU‐RSP conversion tables. However, ionization chamber measurement is required to determine the effective measurement point for each field and to estimate the dose variation. The fluence correction by each depth for scanning proton beams is a simple method and results showed good accuracy. Nevertheless, fluence correction factor requires further investigation because that had up to 1.0% variance as shown in Fig. 4. Heterogeneous phantoms can be effective for evaluating the total dose, including accuracy of dose calculation and machine variations, and can help to identify problems that are not revealed by homogeneous phantom measurements.

## CONFLICT OF INTEREST

The authors have no conflicts of interest directly relevant to the content of this article.

## References

[1] Kanai T , Kawachi K , Kumamoto Y , et al. Spot scanning system for proton radiotherapy. Med Phys. 1980;7:365–369. 6248752 10.1118/1.594693

[2] Zhu XR , Poenisch F , Song X , et al. Patient‐specific quality assurance for prostate cancer patients receiving spot scanning proton therapy using single‐field uniform dose. Int J Radiat Oncol Biol Phys. 2011;81:552–559. 21300457 10.1016/j.ijrobp.2010.11.071

[3] Lomax A . Intensity modulation methods for proton radiotherapy. Phys Med Biol. 1999;44:185–205. 10071883 10.1088/0031-9155/44/1/014

[4] Kooy HM , Grassberger C . Intensity modulated proton therapy. Br J Radiol. 2015;88:20150195. 26084352 10.1259/bjr.20150195 PMC4628542

[5] Lomax AJ . Intensity modulated proton therapy and its sensitivity to treatment uncertainties 2: the potential effects of inter‐fraction and inter‐field motions. Phys Med Biol. 2008;53:1043–1056. 18263957 10.1088/0031-9155/53/4/015

[6] Lomax AJ . Intensity modulated proton therapy and its sensitivity to treatment uncertainties 1: the potential effects of calculational uncertainties. Phys Med Biol. 2008;53:1027–1042. 18263956 10.1088/0031-9155/53/4/014

[7] Ibbott GS , Molineu A , Followill DS . Independent evaluations of IMRT through the use of an anthropomorphic phantom. Technol Cancer Res Treat. 2006;5:481–487. 16981790 10.1177/153303460600500504

[8] Molineu A , Hernandez N , Nguyen T , Ibbott G , Followill D . Credentialing results from IMRT irradiations of an anthropomorphic head and neck phantom. Med Phys. 2013;40:022101. 23387762 10.1118/1.4773309 PMC3555917

[9] Albertini F , Casiraghi M , Lorentini S , Rombi B , Lomax J . Experimental verification of IMPT treatment plans in an anthropomorphic phantom in the presence of delivery uncertainties. Phys Med Biol. 2011;56:4415–4431. 21709345 10.1088/0031-9155/56/14/012

[10] Taylor PA , Kry SF , Alvarez P , et al. Results from the Imaging and Radiation Oncology Core Houston's Anthropomorphic Phantoms Used for Proton Therapy Clinical Trial Credentialing. Int J Radiat Oncol Biol Phys. 2016;95:242–248. 27084644 10.1016/j.ijrobp.2016.01.061 PMC4834872

[11] Mackin D , Zhu XR , Poenisch F , et al. Spot‐scanning proton therapy patient‐specific quality assurance: results from 309 treatment plans. Int J Part Ther. 2014;1:711–720.

[12] Molinelli S , Mairania A , Mirandola A , et al. Dosimetric accuracy assessment of a treatment plan verification system for scanned proton beam radiotherapy: one‐year experimental results and Monte Carlo analysis of the involved uncertainties. Phys Med Biol. 2013;58:3837–3847. 23681116 10.1088/0031-9155/58/11/3837

[13] Zhu X , Li Y , Mackin D , et al. Towards effective and efficient patient‐specific quality assurance for spot scanning proton therapy. Cancers (Basel). 2015;7:631–647. 25867000 10.3390/cancers7020631 PMC4491675

[14] Meier G , Besson R , Nanz A , et al. Independent dose calculations for commissioning, quality assurance and dose reconstruction of PBS proton therapy. Phys Med Biol. 2015;60:2819. 25779992 10.1088/0031-9155/60/7/2819

[15] Mackin D , Li Y , Taylor MB , et al. Improving spot‐scanning proton therapy patient specific quality assurance with HPlusQA, a second‐check dose calculation engine. Med Phys. 2013;40:121708. 24320494 10.1118/1.4828775

[16] White DR , Booz J , Griffith R V. , Spokas JJ , Wilson IJ . ICRU Report 44, Tissue substitutes in radiation dosimetry and measurement. Technical Report (Bethesda, MD) Int. Comm. Radiat. Units Meas; 1989, **os23**(1): NP.

[17] White DR , Griffith R V. , Wilson IJ . ICRU REPORT 46, Photon, Electron, Proton and Neutron Interaction Data for Body Tissues. Technical Report (Bethesda, MD) Int. Comm. Radiat. Units Meas; 1992, **os24**(1): NP.

[18] Schneider U , Pedroni E , Lomax A . The calibration of CT Hounsfield units for radiotherapy treatment planning. Phys Med Biol. 1996;41:111–124. 8685250 10.1088/0031-9155/41/1/009

[19] Grant RL , Summers PA , Neihart JL , et al. Relative stopping power measurements to aid in the design of anthropomorphic phantoms for proton radiotherapy. J Appl Clin Med Phys. 2014;15:4523. 24710437 10.1120/jacmp.v15i2.4523 PMC4283476

[20] Palmans H , Symons JE , Denis J‐M , de Kock EA , Jones DTL , Vynckier S . Fluence correction factors in plastic phantoms for clinical proton beams. Phys Med Biol. 2002;47:3055–3071. 12361210 10.1088/0031-9155/47/17/302

[21] Hirayama S , Takayanagi T , Fujii Y , et al. Evaluation of the influence of double and triple Gaussian proton kernel models on accuracy of dose calculations for spot scanning technique. Med Phys. 2016;43:1437. 26936728 10.1118/1.4942386

[22] Ezzell GA , Burmeister JW , Dogan N , et al. IMRT commissioning: multiple institution planning and dosimetry comparisons, a report from AAPM Task Group 119. Med Phys. 2009;36:5359–5373. 19994544 10.1118/1.3238104

[23] Pflugfelder D , Wilkens JJ , Oelfke U . Worst case optimization: a method to account for uncertainties in the optimization of intensity modulated proton therapy. Phys Med Biol. 2008;53:1689–1700. 18367797 10.1088/0031-9155/53/6/013

[24] Yasui K , Toshito T , Omachi C , et al. A patient‐specific aperture system with an energy absorber for spot scanning proton beams: verification for clinical application. Med Phys. 2015;42:6999–7010. 26632055 10.1118/1.4935528

[25] Toshito T , Omachi C , Kibe Y , et al. A proton therapy system in Nagoya Proton Therapy Center. Austr Phys Eng Sci Med. 2016;39:645–654. 10.1007/s13246-016-0456-8 27271800

[26] Yasui K , Toshito T , Omachi C , et al. Evaluation of dosimetric advantages of using patient‐specific aperture system with intensity‐modulated proton therapy for the shallow depth tumor. J Appl Clin Med Phys. 2018;19:132–137. 29178546 10.1002/acm2.12231 PMC5768032

[27] Sugama Y , Nishio T , Onishi H . Technical note: experimental determination of the effective point of measurement of two cylindrical ionization chambers in a clinical proton beam. Med Phys. 2015;42:3892–3895. 26133590 10.1118/1.4921617

[28] Bhullar AS , Watchman CJ . The effective depth of cylindrical ionization chambers. Phys Med Biol. 2012;273:273–286. 10.1088/0031-9155/57/1/273 22156108

[29] Andreo P , Burns DT , Hohlfeld K , et al. Absorbed Dose Determination in External Beam Radiotherapy. Vienna, Austria: IAEA; 2000, 242.

[30] Arjomandy B , Sahoo N , Zhu XR , et al. An overview of the comprehensive proton therapy machine quality assurance procedures implemented at The University of Texas M. D. Anderson Cancer Center Proton Therapy Center‐Houston. Med Phys. 2009;36:2269–2282. 19610316 10.1118/1.3120288

[31] Zhao L , Das IJ . Gafchromic EBT film dosimetry in proton beams. Phys Med Biol. 2010;55:N291–N301. 20427858 10.1088/0031-9155/55/10/N04

[32] Low DA , Harms WB , Mutic S , Purdy JA . A technique for the quantitative evaluation of dose distributions. Med Phys. 1998;25:656–661. 9608475 10.1118/1.598248

[33] Reinhardt S , Würl M , Greubel C , et al. Investigation of EBT2 and EBT3 films for proton dosimetry in the 4–20 MeV energy range. Radiat Environ Biophys. 2015;54:71–79. 25572031 10.1007/s00411-014-0581-2

[34] Castriconi R , Ciocca M , Mirandola A , et al. Dose–response of EBT3 radiochromic films to proton and carbon ion clinical beams. Phys Med Biol. 2017;62:377–393. 27997377 10.1088/1361-6560/aa5078

